# Adipose tissue characteristics as a new prognosis marker of patients with locally advanced head and neck cancer

**DOI:** 10.3389/fnut.2025.1472634

**Published:** 2025-03-14

**Authors:** Larissa Ariel Oliveira Carrilho, Fabiana Lascala Juliani, Rafaella Caroline de Lellis Moreira, Livia Dias Guerra, Fernanda Silva Santos, Daniela Morais de Holanda Padilha, Sandra Regina Branbilla, Vivian Naomi Horita, Davi Magalhães Leite Novaes, Lígia Macedo Antunes-Correa, Carmem Silvia Passos Lima, Maria Carolina Santos Mendes, José Barreto Campello Carvalheira

**Affiliations:** ^1^Department of Radiology and Oncology, Division of Oncology, School of Medical Sciences, State University of Campinas (UNICAMP), São Paulo, Brazil; ^2^Nestlé Health Science, Vevey, Switzerland; ^3^School of Physical Education (FEF), University of Campinas (UNICAMP), São Paulo, Brazil

**Keywords:** head and neck neoplasms, adipose tissue, body composition, nutrition assessment, malnutrition

## Abstract

**Background:**

Patients with head and neck cancer (HNC) are at increased risk of malnutrition due to the presence of tumor and treatments. Body composition is a prognostic factor in these patients. However, the relationship between adipose tissue characteristics and survival in HNC is still unclear.

**Objective:**

To evaluate the associations of adiposity, the radiodensity of adipose tissue and muscularity with the prognosis of patients with locally advanced HNC undergoing to chemoradiotherapy.

**Methods:**

This retrospective study included 132 patients diagnosed with locally advanced HNC. Body composition assessment was performed using computed tomography (CT) images at the level of the third cervical vertebra (C3). The total adipose tissue radiodensity (TATR), the total adipose tissue index (TATI) and skeletal muscle index (SMI) were evaluated. The primary outcome was overall survival (OS).

**Results:**

Patients in the highest TATI tertile had a lower risk of mortality when compared to those in the lowest tertile, HR: 0.56, 95% confidence Interval (CI): 0.32–0.96; *p* = 0.039. The highest TATR tertile was not associated with death. Patients with greater adiposity had a higher median survival compared to patients with medium and lower TATI (*p* = 0.0193). Individuals with lower TATI had lower energy intake than patients with higher TATI (*p* = 0.03). Additionally, patients with low muscularity had worse OS in the multivariable analysis (HR: 1.77, 95% CI: 1.01–3.07; *p* = 0.044).

**Conclusion:**

In patients with locally advanced HNC, our findings underscore the significance of elevated adiposity, beyond maintained muscularity, as independent protective factors for overall survival. Our study highlights the critical importance of assessing body composition and initiating early nutritional interventions to improve the prognosis of these patients.

## 1 Introduction

Cancer has become a disease with high rates of morbidity and mortality worldwide, with head and neck cancer (HNC) accounting for 337,713 new cases and 177,757 deaths in 2020 (1). Head and neck cancers are tumors originating in the upper aerodigestive tract, including the oral cavity, salivary glands, larynx, nasopharynx, oropharynx, hypopharynx, nasal and paranasal sinuses. Histologically, squamous cell carcinoma (SCC) is the most prevalent type, accounting for approximately 90% of cases, with common sites including the oral cavity, oropharynx, larynx, and hypopharynx (2). HNC is more frequent in male patients, and risk factors include smoking, alcoholism, and exposure to the Human Papillomavirus (HPV) (3–5).

In addition to surgical treatment, radiotherapy, and chemo-radiotherapy are among the commonly used methods for the treatment of locally advanced HNC (6). Those with locally advanced disease can present a variable survival rate of 10–50%, which can be influenced by different factors, such as the performance status of the patient, age, presence of comorbidities, life habits, previous nutritional status and body composition (7, 8).

Patients with HNC have the second highest prevalence of malnutrition among all types of cancer, and involuntary weight loss might be identified in 30–55% of the patients (9–11). This increased risk of malnutrition may occur before diagnosis and may worsen throughout the patient’s journey (9–11). The tumor site and the treatment applied favor the appearance of symptoms that impact the nutritional, such as odynophagia, dysphagia, xerostomia, and dysgeusia (12). This scenario compromises food intake and protein-calorie adequacy, which worsens the risk of malnutrition and other associated disorders, such as sarcopenia, cachexia, and modifications in the adipose tissue (13).

Different methods have been used to assess body composition in cancer, including Bioelectrical Impedance (BIA), Dual-energy X-ray Absorptiometry (DEXA), Magnetic Resonance and Computed Tomography (CT) (14). CT is an exam routinely used in the diagnosis and staging of cancer, which is why it is one of the imaging methods most used in the literature for evaluating body composition in cancer patients (15, 16). The analysis of body composition by CT is commonly performed by assessing the cross-sectional area of body compartments (muscle and adipose tissue) in the region of the third lumbar vertebra (L3) (15). However, images of L3 are not always available in patients with HNC, whereas images of the third cervical vertebra (C3) are more readily accessible.

The assessment of body composition, when added to adequate nutritional intervention, can be associated with a positive outcome in cancer patients (10). Different studies have shown the role of muscularity and adiposity in the prognosis of different oncological populations (17, 18). In HNC, the effect of low muscularity (6, 19) or sarcopenia seems to have a negative association with overall and disease-related survival, post-operative complications, and chemotherapy-related toxicity. In contrast, few studies have evaluated the role of adiposity in patients with HNC, with some showing a favorable prognosis in patients with greater reserves of adipose tissue (20, 21).

In addition to assessing quantity, radiodensity analysis of body compartments is also important and has been explored as a prognostic factor in different pathologies including cancer (22–25). Modifications in adipose tissue radiodensity can be associated with changes in adipose deposits and alterations in the microenvironment and macroenvironment of tumors, which may be related to tumor progression (15, 25). However, the radiodensity of adipose tissue has rarely been explored in the oncological population, and never in the context of HNC. Therefore, the aim of this study was to evaluate the association between total adipose tissue (TAT) characteristics and survival in patients with locally advanced HNC using C3 CT images.

## 2 Methods

### 2.1 Study population

This retrospective study enrolled patients diagnosed with locally advanced HNC between January 2010 and December 2018 at the Hospital de Clínicas – University of Campinas (UNICAMP – Campinas, São Paulo, Brazil).

### 2.2 Inclusion and exclusion criteria

Our sample enrolled all patients diagnosed with HNC with tumor stages III– IVb according to the classification of the American Joint Committee on Cancer (AJCC) cancer staging manual (26), who were starting first line treatment. Eligible criteria also required availability of CT images of the C3 taken within 3 months before the beginning of treatment; information of the date of the last follow-up or death; and comprehensive data on anthropometric, demographic, clinical, and treatment variables available in the medical record.

The exclusion criteria were: patients with primary cancer in other sites at diagnosis, unavailability of CT on the stipulated timeframe, or the presence of low-quality CT images, which would hinder proper body composition analysis.

### 2.3 Data collection

The following variables were collected: sex, age at diagnosis; clinical data (including date of diagnosis and start of treatment), presence of comorbidities; lifestyle habits (such as smoking and alcoholism); weight and height for calculating BMI and body composition variables; tumor topography and staging, concomitant chemotherapy; ECOG (Eastern Cooperative Oncology Group); biochemical tests at diagnosis (neutrophil, lymphocyte counts) for further calculation of the neutrophil-lymphocyte ratio (NLR), an inflammatory marker.

The collected data were recorded in a specific form using the electronic data collection tool—RedCap (26) offered by the Faculty of Medical Sciences at UNICAMP.

### 2.4 Body composition

Body composition was assessed from the retrospective analysis of CT images collected with contrast in the arterial phase that were available in the Arya medical image viewer considering the interval from 3 months before treatment. All analyzed CT images were from exams already performed by patients to assess the stage or progression of the disease, according to medical criteria.

Images of the cross-sectional region of the C3 were evaluated by a trained evaluator. Three raters were included in the analysis of CT’s images. The areas of adipose and muscle tissue (cm^2^) were calculated using the software SliceOMatic V.5.0 (Tomovision, Canada).

The muscle cross-sectional area (CSA) at C3 was used to estimate the CSA of the muscle at L3 using a specific formula created by Swartz et al. (27): CSA at L3 cm^2^ = 27.304 + 1.363 × CSA at C3 (cm^2^) − 0.671 × Age (years) + 0.640 × Weight (kg) + 26.442 × Sex (1 for female and 2 for male). Subsequently, adjusted by height (m^2^), skeletal muscle index (SMI) was calculated as follows: SMI = muscle area in L3 (cm^2^) /height^2^ (m^2^).

**Figure 1:**
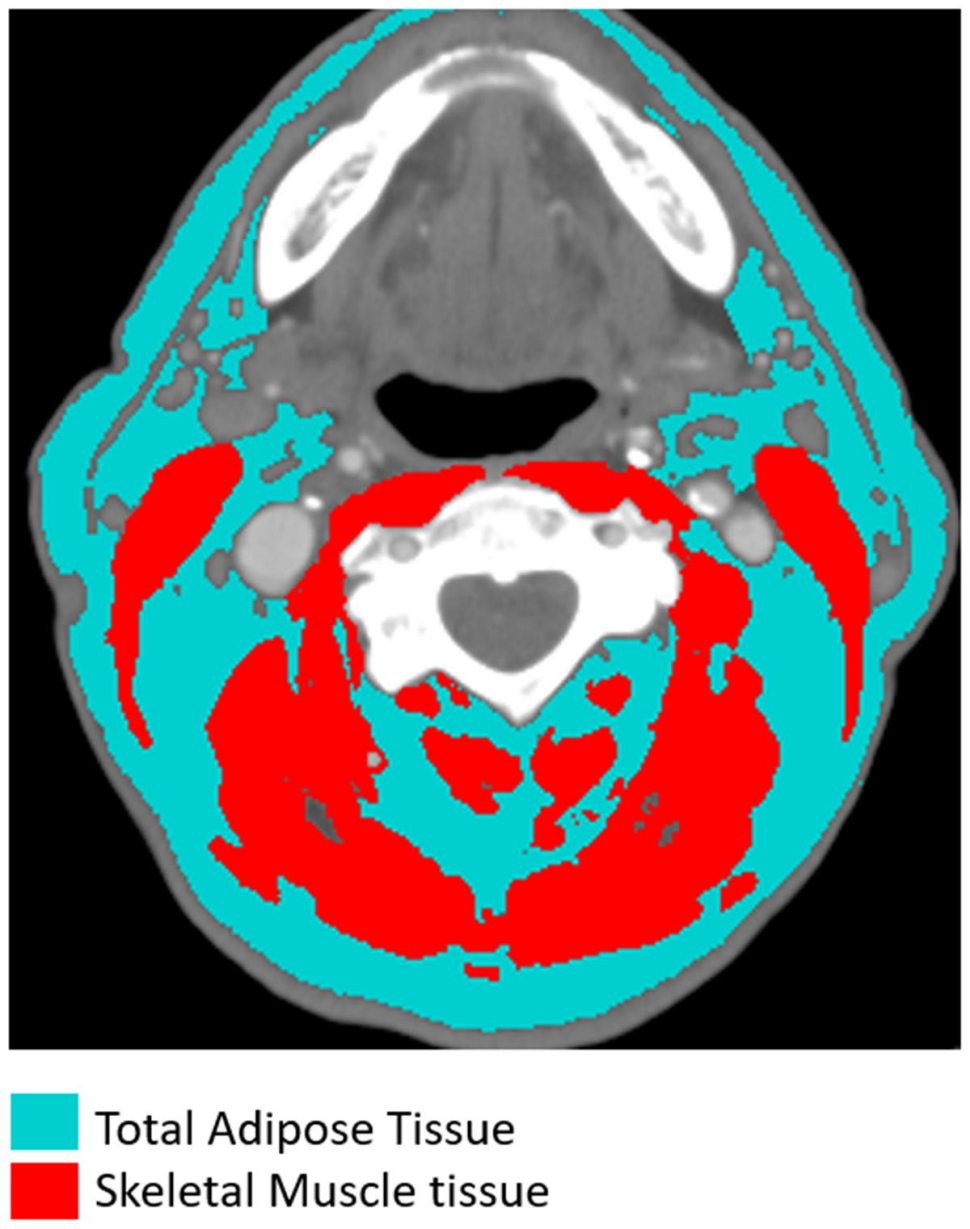
Segmentation of total adipose tissue (with subcutaneous and internal tissues highlighted in blue) and skeletal muscle tissue (including the sternocleidomastoid and paravertebral muscles highlighted in red) at the level of the third cervical vertebra (C3) in CT images.

We considered total adipose tissue (TAT) to include both the subcutaneous and the internal adipose tissue present in the axial section of C3 (Figure 1). The area of adipose tissue was normalized by the height of the individuals to calculate the total adipose tissue index (TATI): TATI = subcutaneous and internal fat area (cm^2^)/height (m^2^). Adipose tissue radiodensity was calculated from the adipose tissue attenuation value, which typically ranges from −190 to −30 Hounsfield Units (28).

### 2.5 Endpoints

The outcome assessment considered the last follow-up date recorded in the medical records or the date of death was considered. Overall survival (OS) was assessed by the time between disease diagnosis and death from any cause.

### 2.6 Ethical aspects

The study was approved by the ethics committee of UNICAMP (CAAE: 42743120.5.0000.5404) and was conducted according to the Helsinki protocol. The patients selected for the study were contacted via telephone (or the family member in case of death) to grant authorization for the research. In case of impossibility of contact, the ethics committee was asked to waive the application of the Free and Informed Consent Form.

### 2.7 Statistical analysis

The Shapiro Wilk test was used to verify the normality of the distributions of the variables. Kruskal-Wallis, chi-squared or Fisher’s Exact statistical tests were applied to investigate differences between groups, when appropriate. Body composition parameters were categorized based on tertiles rather than predefined cut-off values. OS was assessed by Kaplan–Meier curves, and comparisons between groups were performed using the log-rank test. Univariate and multivariate survival analyzes were conducted using Cox proportional hazard regression. Continuous variables are presented as medians and interquartile ranges and as frequency if categorical. A significance level of 5% was considered. Statistical analyzes were performed using Stata software version 17.0 (StataCorp LP^®^).

## 3 Results

### 3.1 Demographic, clinical, and laboratory findings

**Figure 2:**
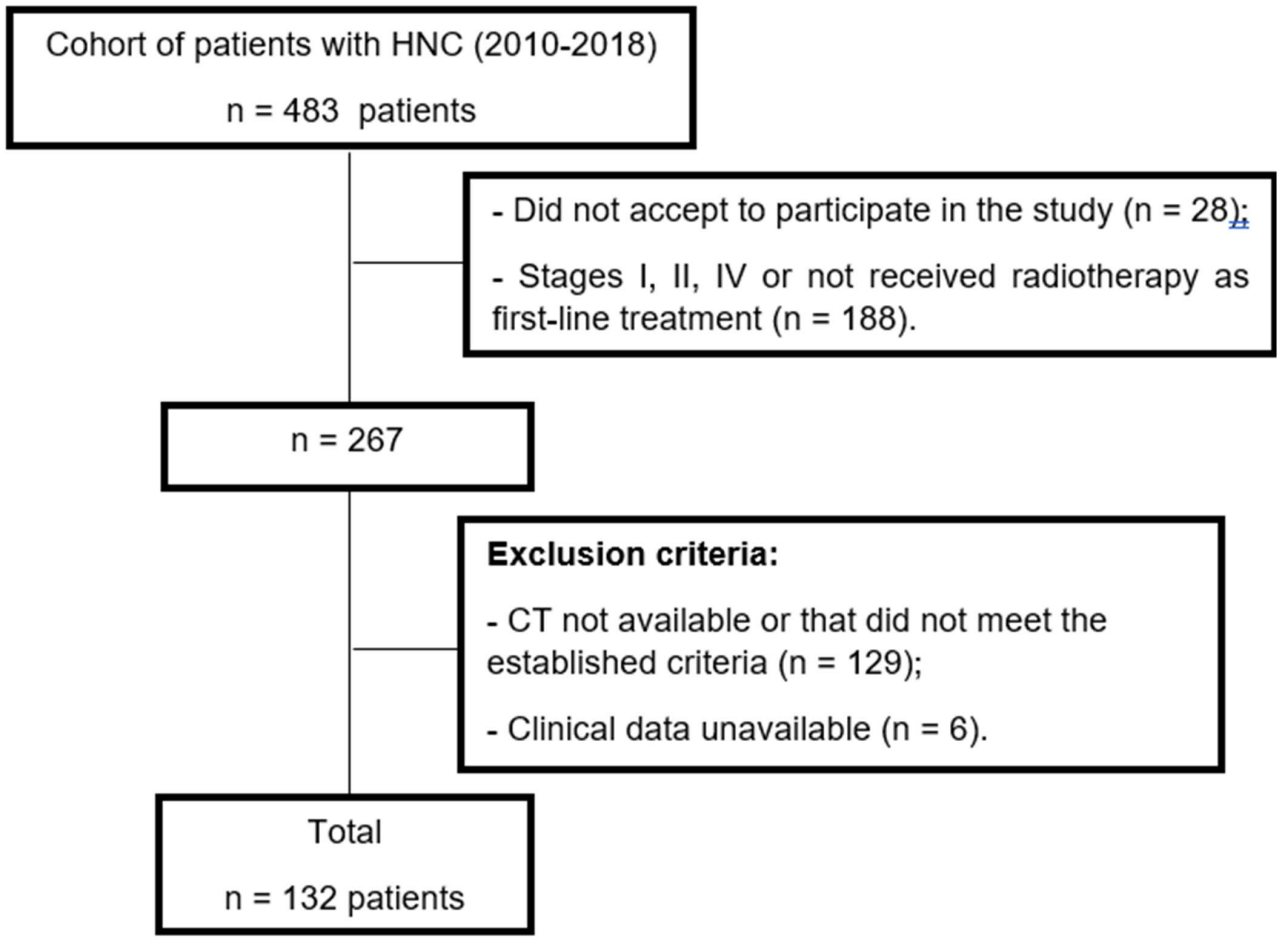
Patients selection flowchart for this study.

A total of 132 patients were included in the study (Figure 2). Demographic and clinical characteristics are presented according to the tertiles of TATI in Table 1. Among the subgroups, the sample was homogeneous in terms of sex, alcohol consumption, tumor topography, staging and functionality assessed by ECOG. There was a greater predominance of male individuals (87.9%), aged between 55 and 70 years (60.6%) and considered eutrophic according to the BMI (52.3%). Regarding anatomical location, the most frequent tumors in the sample were oral cavity (31.1%) and larynx (39.4%). The majority of the sample (92.4%) underwent radiotherapy in combination with chemotherapy, primarily with cisplatin (83.5%) or carboplatin (15.7%). Additionally, 8.6% received radiotherapy alone.

**Table 1:** Selected demographic and clinical characteristics and laboratory findings according to total adipose tissue index of patients with locally advanced head and neck cancer.

		Total Adipose Tissue Index (TATI)			
Characteristic	All-patients, *n* = 132	Low tertile, *n* = 43 Female: 1.7–5.8 Male: 0.0–5.1	Middle tertile, *n* = 44 Female: 8.0–12.7 Male: 5.4–10.1	High tertile, *n* = 45 Female: 15.3–21.2 Male: 10.1–24.9	*p* value
Age, no. (%)					
< 55	35 (26.5)	12 (27.9)	9 (20.5)	14 (31.1)	0.029^a^
55–70	80 (60.6)	30 (69.8)	29 (65.9)	21 (46.7)	
>70	17 (12.9)	1 (2.3)	6 (13.6)	10 (22.2)	
Sex, no. (%)					
Male	116 (87.9)	38 (88.4)	39 (88.6)	39 (86.7)	1.000^a^
Female	16 (12.1)	5 (11.6)	5 (11.4)	6 (13.3)	
BMI, no. (%)					
<18.5	30 (22.7)	23 (53.5)	7 (15.9)	0 (0.0)	<0.001^a^
18.5–24.9	69 (52.3)	19 (44.2)	30 (68.2)	20 (44.4)	
>25	33 (25.0)	1 (2.3)	7 (15.9)	25 (55.6)	
Smoking status, no. (%)					
Never	10 (7.6)	1 (2.3)	4 (9.1)	5 (11.1)	0.042^a^
Former (more than 5 years)	69 (52.3)	17 (39.5)	25 (56.8)	27 (60.0)	
Active	53 (40.1)	25 (58.1)	15 (34.1)	13 (28. 9)	
Alcohol consumption, no. (%)					
Never	17 (12.9)	4 (9.3)	4 (9.1)	9 (20.0)	0.190^a^
Former (more than 5 years)	82 (62.1)	24 (55.8)	29 (65.9)	29 (64.4)	
Active	33 (25.0)	15 (34.9)	11 (25.0)	7 (15.6)	
Hypertension, no. (%)	38 (28.8)	2 (4.7)	16 (36.4)	20 (44.5)	<0.001^a^
Diabetes, no. (%)	14 (10.6)	2 (4.7)	5 (11.4)	7 (15.6)	0.273^a^
Topography, no. (%)					
Oral Cavity	41 (31.1)	15 (34.9)	13 (29.5)	13 (28.9)	0.146^b^
Oropharynx	39 (29.5)	17 (39.5)	13 (29.5)	9 (20.0)	
Larynx	52 (39.4)	11 (25.6)	18 (40.9)	23 (51.1)	
Stage, no. (%)					
III	27 (20.5)	7 (16.3)	6 (13.6)	14 (31.1)	0.274^a^
IVA	75 (56.8)	24 (55.8)	28 (63.6)	23 (51.1)	
IVB	30 (22.7)	12 (27.9)	10 (22.7)	8 (17.8)	
ECOG, no. (%)					
0–1	121 (91.7)	40 (93.0)	38 (86.4)	43 (95.6)	0.301^a^
2–3	11 (8.3)	3 (7.0)	6 (13.6)	2 (4.4)	
Induction chemotherapy, no. (%)	18 (13.7)	8 (18.6)	1 (2.3)	9 (20.5)	0.016^a^
Concomitant chemotherapy, no. (%)^c^	122 (92.4)	38 (88.4)	41 (93.2)	43 (95.6)	0.383^a^

a Fisher’s exact test.

b Chi-squared test.

c Cisplatin (83.5%) or carboplatin (15.7%).

Patients with a higher TATI were older (22%; *p* = 0.029), had a lower frequency of smoking (28.9%; *p* = 0.042) and a trend toward lower frequency of alcoholism (15.6%; *p* = 0.190). Additionally, they had a higher prevalence of overweight (55.6%; *p* < 0.001), systemic arterial hypertension (44.5%*p* < 0.01) and a similar prevalence of diabetes (15.6%; *p* = 0.273).

According to the adequacy of muscularity, 107 (81%) patients had normal muscularity and 25 (19%) had low muscularity. Patients with low muscularity had a lower BMI (76% vs. 10.3%; *p* < 0.001) and were more classified with ECOG 2 and 3 (20% vs. 5.6%, *p* = 0.034), indicating worse functionality (Supplementary Table 1).

### 3.2 Univariate and multivariable analysis of body composition and prognosis

Table 2 shows the results of the age-adjusted (Model A) and multivariate analyses (Model B) for adiposity according to the adjusted models and overall survival (OS). In Model B, TATI and TATR were adjusted for age, ECOG, diabetes, hypertension, concomitant chemotherapy, and tumor stage.

**Table 2:** Univariate and multivariable models for adiposity.

				Model A		Model B^a^	
				Age adjusted		Multivariable adjusted	
Characteristic	Patients at risk	Number of events	Median OS (months)	HR (95% CI)	P_trend_	HR (95% CI)	P_trend_
TATI							
Low (tertile 1)	43	38	13.9	1 [Reference]	**0.007**	1 [Reference]	**0.039**
Middle (tertile 2)	44	29	22.6	0.54 (0.33–0.88)		0.54 (0.32–0.92)	
High (tertile 3)	45	31	27.9	0.49 (0.30–0.79)		0.56 (0.32–0.96)	
TATR							
Low (tertile 1)	39	26	32.1	1 [Reference]	**0.022**	1 [Reference]	0.105
Middle (tertile 2)	39	29	22.9	1.66 (0.96–2.85)		1.56 (0.90–2.70)	
High (tertile 3)	40	31	17.0	2.15 (1.24–3.70)		1.81 (1.02–3.23)	

a Model B is adjusted for age (categorical), ECOG (categorical), diabetes (binary), hypertension (binary), concomitant chemotherapy (binary), stage (categorical).

TATI, Total Adipose Tissue Index; TATR, Total Adipose Tissue Radiodensity.

In Model A, it was observed that patients with a higher tertile of TATI had a lower risk of death (HR: 0.49; 95% CI: 0.30–0.79; *p* = 0.007). Conversely, higher radiodensity in adipose tissue was significantly associated with an increased risk of mortality (HR: 2.15; 95% CI: 1.24–3.70; *p* = 0.022).

In Model B, patients in the highest TATI tertile maintained a lower risk of mortality (HR: 0.56; 95% CI: 0.32–0.96; *p* = 0.039). Contrary to what was observed with radiodensity in Model A, patients with higher TATR had no significant association with the risk of death in Model B (*p* = 0.105). However, when comparing the first and third tertiles, Model B indicated that individuals in the highest tertile of TATR had a significantly increased risk of mortality (HR: 1.81; 95% CI: 1.02–3.23).

**Figure 3:**
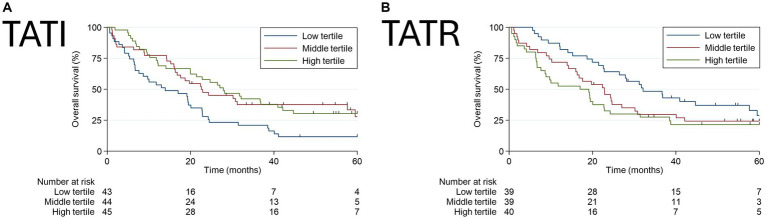
Evaluation of Overall Survival using Kaplan-Meier curves according to **(A)** Total Adipose Tissue Index (TATI) and **(B)** Total Adipose Tissue Radiodensity (TATR).

Figure 3 presents the Kaplan–Meier curves illustrating the overall survival probabilities based on adiposity characteristics, as analyzed in Models A and B.

Supplementary Table 2 reports the multivariate analysis according to muscularity for the same adjusted models for adiposity (A and B) according to OS. We identified a higher risk of death in Model A (HR: 2.11, 95% CI: 1.27–3.52; *p* = 0.004) and Model B for those with low muscularity (HR: 1.77, 95% CI: 1.01–3.07; *p* = 0.044).

Supplementary Figure 1 shows the Kaplan–Meier curves illustrating the overall survival probabilities based on the adequacy of muscularity, as analyzed in Models A and B.

### 3.3 Evaluation of the inflammation and food intake

The assessment of the inflammatory profile by the NRL did not show a significant association with the adiposity evaluated by the TATI (*p* = 0.47) or with muscularity (*p* = 0.20).

**Figure 4:**
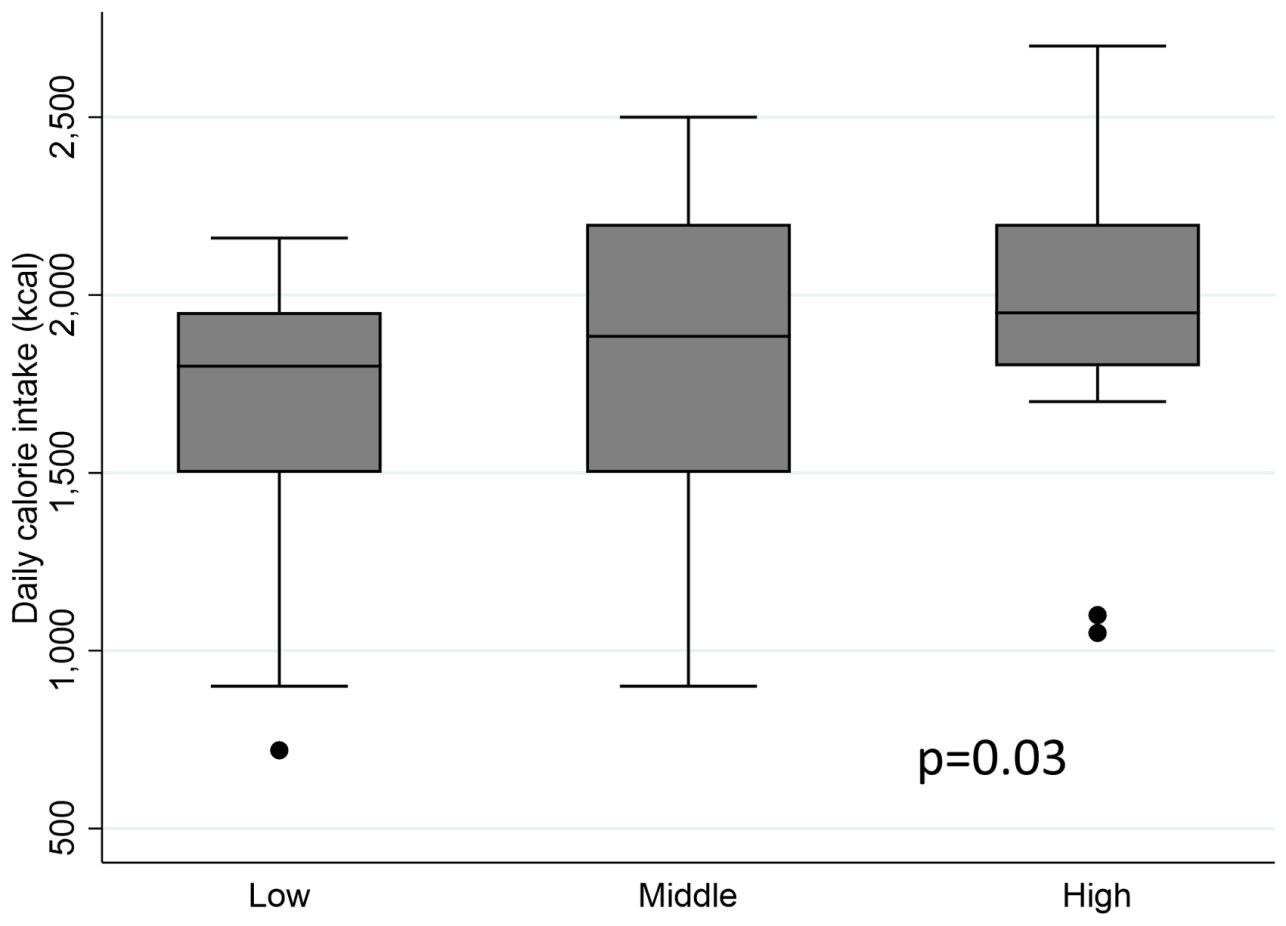
Analysis of energy intake according to adiposity tertile.

Individuals with a lower amount of total adipose tissue (TAT) had lower energy intake than patients with a higher TATI (1800 kcal versus 1950 kcal; *p* = 0.03) (Figure 4). Additionally, patients in the high-TATI tertile had a higher energy requirement compared to those in the lower tertile (*p* = 0.004). In Cox analyses, weight loss showed no significant association with survival (*p* = 0.44).

## 4 Discussion

Our findings suggest that greater adiposity and lower radiodensity of adipose tissue may be protective factors for OS in patients with locally advanced HNC. Additionally, low muscularity is identified as a poor prognostic factor for survival in patients with locally advanced HCN. However, weight loss and inflammation did not show prognostic significance in this population.

The effects of overweight and obesity on prognosis in oncological populations vary and can differ based on the type of tumor. For example, higher adiposity has been associated with a higher incidence and progression of different tumors such as colon, breast and pancreas (29–32). However, in HNC, the role of obesity and adipose tissue has not yet been fully elucidated. In our population, patients in the higher adiposity tertile had more hypertension, diabetes and excess weight according to BMI. Interestingly, greater adiposity had a protective role in OS in our cohort, in which individuals with a higher TATI tertile and a higher BMI had a better prognosis. A similar association was observed by Hicks et al. (29), who reported that HNC patients undergoing radiotherapy and with higher BMI had better OS (*p* < 0.001) and recurrence-free survival (*p* < 0.001). The same was identified by Zhao et al. (33) and Grossberg et al. (34), although, in the latter study, the association was not significant. Altogether, these results suggest that the notion of a higher BMI reflecting energy reserves—and thus being associated with better survival outcomes in HNC patients compared to those with normal or low BMI, a phenomenon known as the “obesity paradox”—warrants further examination. Notably, although adiposity may serve as an energy and metabolic reservoir that provides some protection in HNC patients, the presence of low muscularity, or sarcopenia, has a significantly negative impact on prognosis, highlighting the importance of separately evaluating body compartments.

Although positive associations were found between BMI and survival in HNC, BMI is known to be a limiting factor in the assessment of body composition, as it does not provide a thorough analysis of different body compartments, such as adipose tissue and skeletal muscle mass (29, 34, 35). In our cohort, patients with low muscularity had a worse functionality, evidenced by the ECOG status, and a poor OS in the multivariable analysis. Kubrak et al. (16) analyzed a cohort of 1,231 patients and reported that the subpopulation of patients with locally advanced HNC (*n* = 664) with moderate and severe SMI depletion had an increased risk of death (*p* < 0,001). Consistently, Bentahila et al. (36) evaluated the impact of low muscularity on survival and treatment tolerance in HNC patients and concluded that patients with low muscularity had a worse disease free survival (HR 2.174; *p* = 0.0001), but the inadequacy of skeletal muscle was not associated with less tolerance of oncological treatment.

Many studies have predominantly focused the assessment of body composition in HNC to muscularity. However, the evaluation of adipose tissue’s role in these populations remains minimally explored (36–38). Consistent with our results obtained using a C3 CT image, a retrospective study with 881 patients undergoing curative radiotherapy identified that a higher subcutaneous adipose tissue index had a protective association with distant metastasis-free survival (HR: 0.65; *p* = 0.015), locoregional control (HR: 0.758; *p* = 0.047) and OS (HR: 0.604; *p* < 0.001) (39). He et al. (20) evaluated patients with non-metastatic nasopharyngeal cancer and found a negative association between survival and lower visceral adiposity (*p* < 0.001, HR, 1.884; 95% CI, 1.436–2.473) and lower subcutaneous adiposity (*p* = 0.022, HR, 1.334, 95% CI, 1.043–1.706). Moreover, Lee and colleagues (21) reported a similar result regarding survival and visceral fat stores.

A prospective study that evaluated the nutritional consequences of radiotherapy in patients with nasopharyngeal cancer and evaluated body composition by DEXA described that there was a significant loss in adipose tissue at different times after radiotherapy, and that after 6 months of treatment, the stores of adipose tissue continued to decrease, while lean mass remained virtually unchanged (40). In other populations, such as those with metastatic colorectal cancer, greater subcutaneous adiposity was also protective for mortality (HR, 0.51; 95% CI, 0.29_0.88; Ptrend <0.025) (41). However, in patients with non-metastatic breast cancer, the highest adiposity tertile was negative for survival (23). Altogether, these findings underscore the importance of maintaining adequate adipose tissue and skeletal muscle mass during and after treatment for patients with HNC.

The assessment of adipose tissue radiodensity was a predictive factor in the prognosis in our population in age-adjusted model, and in the comparison between the low and high tertiles in multivariable adjusted analysis (Model B). However, the trend across tertiles was not observed in Model B. The higher attenuation of adipose tissue can be attributed to different factors that normally indicate a change in metabolism in the tissue, such as greater deposition of fibroblasts, greater vascularization, increased water content and lower lipid content (18). Lee et al. (21) found that patients with HNC who had low visceral adiposity and higher Visceral Adiposity Radiodensity (VATR) had worse progression free survival and distant metastasis-free survival compared to those with high visceral adipose tissue (VAT) and lower attenuation. Previous articles from our group showed similar associations of radiodensity in different populations, such as multiple myeloma and metastatic colorectal cancer patients (18, 41). The same was found in hepatocellular carcinoma (42) and esophageal cancer (43). These findings suggest that higher adipose radiodensity may be a negative prognostic in cancer.

The increase in adipose tissue radiodensity may be associated with the browning of white adipocytes, a process linked to cachexia. This phenomenon may be triggered by factors such as increased inflammatory infiltrates in adipose tissue, energy restriction, and significant weight loss (18, 41). Zoabi et al. observed that individuals with higher BMI had lower adipose tissue radiodensity (25). Furthermore, the study reported substantial differences in adipose tissue composition associated with changes in radiodensity. Adipose tissue with lower radiodensity had a higher concentration of short-chain fatty acids, while tissue with higher radiodensity exhibited greater abundance of lipids such as various phospholipids, ceramides, cholesterol esters, and diglycerides. Individuals with higher adipose tissue radiodensity also showed more pronounced adipocyte atrophy and greater tissue inflammation.

It is suggested that advanced-stage tumors may present greater release of inflammatory factors contributing to more pronounced lipolysis (44). Additionally, this may be linked to the dose of radiotherapy applied, as the affected area is typically exposed to a limited radiation dose. However, the relationship between body composition and inflammation in head and neck cancer is still unclear. In our study, there was no association between adiposity or muscularity and inflammation, evaluated by the NLR. On the contrary, in a different cohort of HNC patients, those with low visceral adipose tissue had higher NLR (*p* = 0.028) (20). Another previous study in locally advanced HNC identified that patients with higher NLR and sarcopenia had a worse OS (HR: 2.78; CI 95%: 1.67–4.63; *p* < 0.001) and progression free survival (HR: 2.14; CI 95%:1.42–3.22; *p* < 0.001) in the multivariable analysis (45). Our data reinforce the hypothesis that in our patients, the loss of adipose tissue may not be due to tumor inflammation, but rather to a possible process of primary malnutrition associated with negative energy balance, since the average energy intake was lower in patients with lower tertile of adiposity and no significant associations were found related to weight loss and survival. Given that we examined adipose tissue in the C3 region, another possibility is that different adipose tissue compartments may uniquely reflect the inflammatory milieu.

Evidence underscores the role of adipose tissue in cancer aggressiveness. First, during cancer progression, tumor cells engage in metabolic symbiosis with adjacent adipose tissue (46). Second, browning of adipose tissue activates angiogenesis, increasing vascularity and promoting tumor growth (46, 47). Third, adipose tissue surrounding tumors interacts with tumor cells through cytokine signaling, fostering a pro-inflammatory environment (48). Lastly, reduced adiponectin secretion from peritumoral adipose tissue is linked to inflammation, heightened tumor aggressiveness, and increased metastatic potential. Thus, the reduced adipose tissue and increased radiodensity in the C3 region observed herein may not only result from tumor-induced cachexia but could also modulate tumor growth in a vicious cycle.

Weight loss in patients with HNC is very common and is usually present at the time of diagnosis, often being associated with nutritional symptoms produced by the tumor and the treatment, which compromises food intake (11, 49). Regarding to caloric adequacy, Kubrak et al. evaluated the clinical determinants of weight loss in HNC cancer, and observed that low caloric intake in patients with HNC who were undergoing chemoradiotherapy and whose weight loss was consistent with a total energy deficit and symptoms associated with eating were significant predictors of weight loss (49). As mentioned, in our sample, weight loss had no significant association with survival, and similar findings were identified by Zhuang et al. (50). Contrary to expectations, Grossberg and colleagues observed that in patients with locoregional HNC, weight loss >5% tended improve survival (HR, 0.48; 95% CI, 0.21–1.28; *p* = 0.09) (34).

Despite these different findings, it is already well established that malnutrition can negatively impact the outcomes of patients with cancer, where nutrition and oncology guidelines reinforce the importance of early screening of malnutrition, with emphasis on nutrition being included at the beginning of the cancer patient’s journey, already at the time of diagnosis (13, 51). Once malnutrition or nutritional risk is detected, an assessment of the nutritional status should be carried out, prioritizing the evaluation of body composition and the inclusion of nutritional therapy to complement the caloric-protein intake (13, 52). Although our sample had limited information on patients who received nutritional therapy, Bargetzi et al. identified that nutritional therapy can reduce mortality in cancer patients with a higher risk of malnutrition. In general, this finding reinforces the importance of the nutritional intake of these patients for positive changes in outcomes (53).

The limitations of our study include its retrospective design, the lack of data on early nutritional screening and supportive nutritional therapy, and the constraints related to the sample size and composition. In addition, although variations in contrast phases may introduce variability in adipose tissue radiodensity, it is unlikely that contrast affected our results. This is due to the standardized techniques employed at our institution, which typically uses contrast-enhanced CT scans in the arterial phase for patients with HNC (54). Finally, this study is the first in the literature to assess the internal and subcutaneous fat in the C3 region in patients with HNC. This may provide more reliable data on the relationship between the total C3 fat compartment and survival.

## 5 Conclusion

Our results suggest that higher adiposity and normal muscularity are independent protective factors for overall survival in patients with locally advanced HNC treated with chemoradiotherapy. The assessment of body composition, combined with early nutritional intervention, and the preservation of muscle mass and adipose tissue, may play a role in improving the outcomes of locally advanced HNC patients undergoing radiotherapy.

## Data Availability

The raw data supporting the conclusions of this article will be made available by the authors, without undue reservation.

## Glossary

AJCC: American Joint Committee on Cancer
BIA: Bioelectrical Impedance
BMI: Body Mass Inde
CSA: Cross-Sectional Area
CT: Computed Tomography
CI: Confidence Interval
DFS: Disease-Free Survival
DEXA: Dual-Energy X-Ray Absorptiometry
ECOG: Eastern Cooperative Oncology Group
HR: Hazard Ratio
HNC: Head and Neck Cancer
HPV: Human Papillomavirus
NLR: Neutrophil Lymphocyte Ratio
OS: Overall Survival
SMI: Skeletal Muscle Index
TAT: Total Adipose Tissue
TATI: Total Adipose Tissue Index
TATR: Total Adipose Tissue Radiodensity
C3: Third Cervical Vertebra
L3: Third Lumbar Vertebra
VATR: Visceral Adiposity Radiodensity
VAT: Visceral Adipose Tissue
