# Supplementary material for: Adipose tissue characteristics as a new prognosis marker of patients with locally advanced head and neck cancer
Source: Front Nutr. 2025 Mar 14;12:1472634. doi: 10.3389/fnut.2025.1472634 (PMC11949816; doi:10.3389/fnut.2025.1472634)
Supplement: Supplementary file 1 [file Image_1.pdf]

Supplementary Data

Figure 2A. Association between NLR and adiposity

```
. kwallis nlr , by (t_sfi)

Kruskal-Wallis equality-of-populations rank test
```

t_sfi	Obs	Rank sum
Low	37	2111.00
Middle	41	2679.00
High	41	2350.00

```
  chi2(2) =  1.501
     Prob = 0.4722

  chi2(2) with ties =  1.501
               Prob = 0.4722
```

Figure 2B. Association between NLR and muscularity

```
Two-sample Wilcoxon rank-sum (Mann-Whitney) test
```

sarcopenia	Obs	Rank sum	Expected
No	96	5570	5760
Yes	23	1570	1380
Combined	119	7140	7140

```
Unadjusted variance    22080.00
Adjustment for ties       -0.31
                     ----------
Adjusted variance      22079.69

H0: nlr(sarcop~a==No) = nlr(sarcop~a==Yes)
         z = -1.279
Prob > |z| = 0.2010
Exact prob = 0.2036
```
