# Supplementary material for: Adipose tissue characteristics as a new prognosis marker of patients with locally advanced head and neck cancer
Source: Front Nutr. 2025 Mar 14;12:1472634. doi: 10.3389/fnut.2025.1472634 (PMC11949816; doi:10.3389/fnut.2025.1472634)

Figure 1A. Evaluation of Overall Survival using Kaplan-Meier curves according to Muscularity

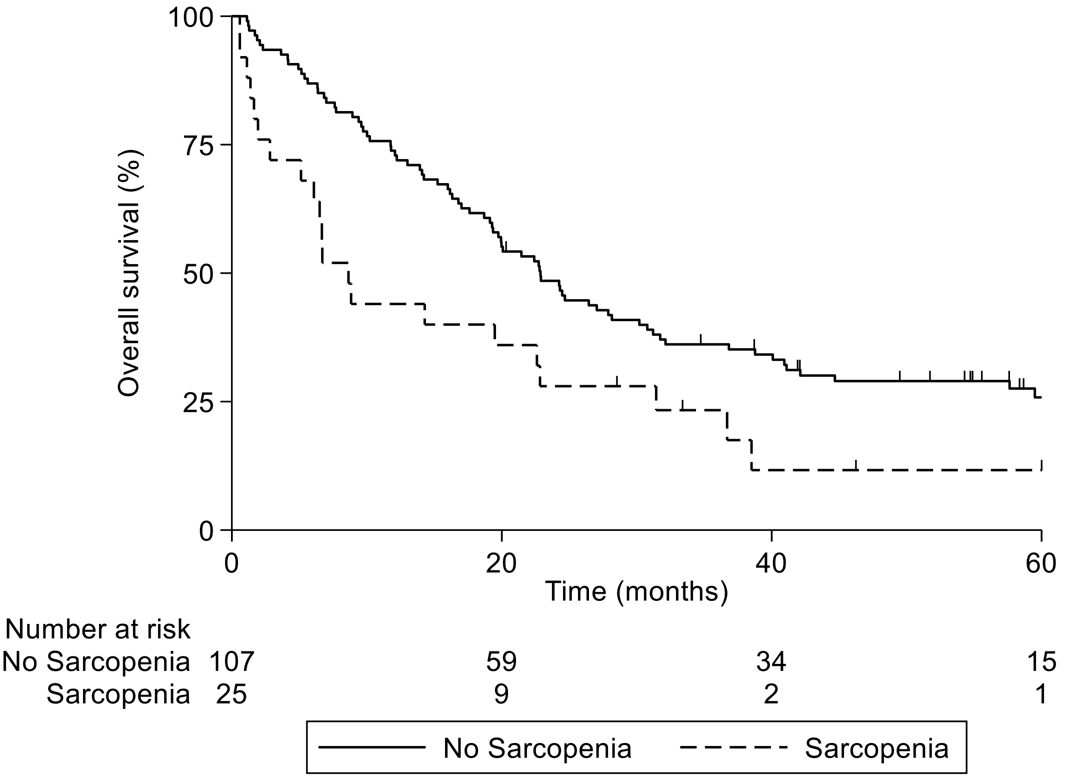

Figure 1B. Evaluation of Disease-Free Survival using Kaplan-Meier curves according to Muscularity

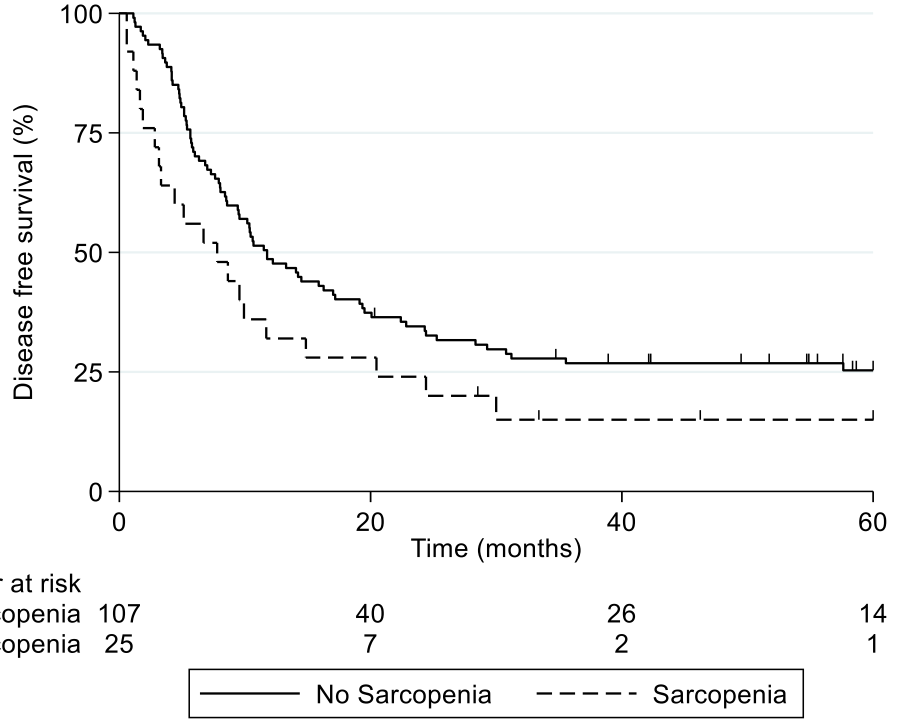

Supplement: Supplementary file 2 [file Image_2.pdf]
