# Supplementary material for: Adipose tissue characteristics as a new prognosis marker of patients with locally advanced head and neck cancer
Source: Front Nutr. 2025 Mar 14;12:1472634. doi: 10.3389/fnut.2025.1472634 (PMC11949816; doi:10.3389/fnut.2025.1472634)
Supplement: Supplementary file 3 [file Table_1.docx]

**Supplementary Data**

**Table 1.** Demographic and Clinical Characteristics based on Muscularity in Patients with Locally Advanced Head and Neck Cancer undergoing Chemoradiotherapy.

| Characteristic | All-patients, n=132 | Normal Muscularity | Low Muscularity | P value |
| --- | --- | --- | --- | --- |
| Age, № (%) |  |  |  |  |
| <55 | 35 (26.5) | 32 (29.9) | 3 (12.0) | 0.116^a^ |
| 55-70 | 80 (60.6) | 63 (58.9) | 17 (68.0) |  |
| >70 | 17 (12.9) | 12 (11.2) | 5 (20.0) |  |
| Sex, № (%) |  |  |  |  |
| Male | 16 (12.1) | 12 (11.2) | 4 (16.0) | 0.504^a^ |
| Female | 116 (87.9) | 95 (88.8) | 21 (84.0) |  |
| BMI, № (%) |  |  |  |  |
| <18.5 | 30 (22.7) | 11 (10.3) | 19 (76.0) | <0.001^a^ |
| 18.5-24.9 | 69 (52.3) | 64 (59.8) | 5 (20.0) |  |
| >25 | 33 (25.0) | 32 (29.9) | 1 (4.0) |  |
| Smoking status, № (%) |  |  |  |  |
| Never smoker | 10 (7.6) | 9 (8.4) | 1 (4.0) | 0.091^a^ |
| Former smoker (more than 5 years) | 69 (52.3) | 51 (47.7) | 18 (72.0) |  |
| Active smoker | 53 (40.1) | 47 (43.9) | 6 (24.0) |  |
| Alcohol consumption, № (%) |  |  |  |  |
| Never | 17 (12.9) | 14 (13.1) | 3 (12.0) | 0.190^a^ |
| Former (more than 5 years) | 82 (62.1) | 64 (59.8) | 18 (72.0) |  |
| Active | 33 (25.0) | 29 (27.1) | 4 (16.0) |  |
| Hypertension, № (%) | 38 (28.8) | 32 (29.9) | 6 (24.0) | 0.631^a^ |
| Diabetes, № (%) | 14 (10.6) | 13 (12.2) | 1 (4.0) | 0.468^a^ |
| Topography, № (%) |  |  |  |  |
| Oral Cavity | 41 (31.1) | 31 (29.0) | 10 (40.0) | 0.239^a^ |
| Oropharynx | 39 (29.5) | 35 (32.7) | 4 (16.0) |  |
| Larynx | 52 (39.4) | 41 (38.3) | 11 (44.0) |  |
| Stage, № (%) |  |  |  |  |
| III | 27 (20.5) | 23 (21.5) | 4 (16.0) | 0.910^a^ |
| IVA | 75 (56.8) | 60 (56.1) | 15 (60.0) |  |
| IVB | 30 (22.7) | 24 (22.4) | 6 (24.0) |  |
| ECOG, № (%) |  |  |  |  |
| 0-1 | 121 (91.7) | 101 (94.4) | 20 (80.0) | 0.034^a^ |
| 2-3 | 11 (8.3) | 6 (5.6) | 5 (20.0) |  |
| Induction chemotherapy, № (%) | 18 (13.7) | 15 (14.2) | 3 (12.0) | 1.000^a^ |
| Concomitant chemotherapy, № (%) | 122 (92.4) | 102 (95.3) | 20 (80.0) | 0.021^a^ |

Abbreviations: BMI: Body mass index; ECOG: Eastern Cooperative Oncology Group performance scale

^a^Fisher’s exact test
