# Supplementary material for: Adipose tissue characteristics as a new prognosis marker of patients with locally advanced head and neck cancer
Source: Front Nutr. 2025 Mar 14;12:1472634. doi: 10.3389/fnut.2025.1472634 (PMC11949816; doi:10.3389/fnut.2025.1472634)
Supplement: Supplementary file 4 [file Table_2.docx]

**Supplementary Data**

**Table 2.** Univariate and Multivariable Models for Muscularity

|  |  |  |  | **Model A** |  | **Model B^a^** |  |
| --- | --- | --- | --- | --- | --- | --- | --- |
|  |  |  |  | **Age adjusted** | | **Multivariable adjusted** | |
|  |  |  |  |  |  |  |  |
| **Characteristic** | **Patients**  **at risk** | **Number of**  **events** | **Median OS**  **(months)** | **HR (95% CI)** | **P** | **HR (95% CI)** | **P** |
| **Disease free survival** |  |  |  |  |  |  |  |
| Normal muscularity | 107 | 79 | 11.8 | 1 [Reference] | **0.008** | 1 [Reference] | 0.052 |
| Low muscularity | 25 | 22 | 7.8 | 1.97(1.20–3.24) |  | 1.70(1.00–2.92) |  |
| **Overall Survival** |  |  |  |  |  |  |  |
| Normal muscularity | 107 | 77 | 22.9 | 1 [Reference] | **0.004** | 1 [Reference] | **0.044** |
| Low muscularity | 25 | 21 | 8.6 | 2.11(1.27–3.52) |  | 1.77(1.01–3.07) |  |

Abbreviations: CI: confidence interval.

^a^Model B is adjusted for age (categorical), ECOG (categorical), diabetes (binary), hypertension (binary), concomitant chemotherapy (binary), stage (categorical)
